# GIST-ery solved: story of an intestinal mass in a young lady

**DOI:** 10.1093/jscr/rjae687

**Published:** 2024-11-07

**Authors:** Shikha Jayasheelan, Saraswathy Sreeram, Akash NS, Abhay Mohan

**Affiliations:** Department of Pathology, Kasturba Medical College, Mangalore, Manipal Academy of Higher Education, Manipal 576 104, Karnataka, India; Department of Pathology, Kasturba Medical College, Mangalore, Manipal Academy of Higher Education, Manipal 576 104, Karnataka, India; Department of Pathology, Kasturba Medical College, Mangalore, Manipal Academy of Higher Education, Manipal 576 104, Karnataka, India; Department of Pathology, Kasturba Medical College, Mangalore, Manipal Academy of Higher Education, Manipal 576 104, Karnataka, India

**Keywords:** GIST, CD117, immunohistochemistry, CFT

## Abstract

A 19-year-old woman presented with abdominal pain and a palpable mass, initially suspected to be a gastrointestinal stromal tumor (GIST) based on imaging. Surgical excision revealed a sclerotic spindle cell neoplasm with minimal cytological atypia, but immunohistochemistry (IHC) was negative for GIST-specific markers. The pan-negative IHC profile, along with calcification foci and low Ki67 index (<1%), led to a diagnosis of calcifying fibrous tumor (CFT). This case highlights the importance of precise diagnostic evaluation and consideration of rare entities like CFT. Comprehensive histopathological evaluation and IHC are essential diagnostic tools, as they can distinguish between GIST and CFT, leading to accurate treatment and patient management. This case underscores the value of thorough pathological assessment in resolving diagnostic challenges.

## Introduction

Calcifying fibrous tumor (CFT) is a rare benign mesenchymal neoplasm characterized by distinctive histopathological features, including hyalinized collagenous fibrous tissue with psammomatous and dystrophic calcifications [1]. In the past, CFT has been recorded in the gastrointestinal tract, abdominal cavity, pleura and neck in addition to its original description as a subcutaneous soft tissue entity [2]. Accurate diagnosis through detailed histopathological and immunohistochemical evaluation is crucial, as its presentation can mimic more common neoplasms such as gastrointestinal stromal tumors (GISTs).

## Case report

**Figure 1:**
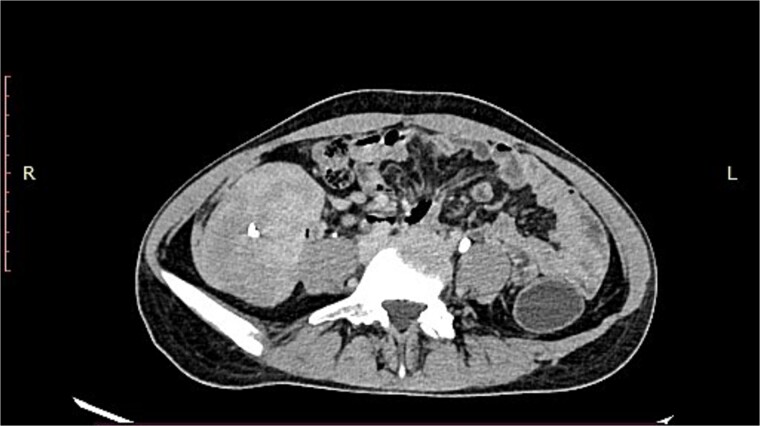
CT image showing a well-defined smooth lobulated heterogeneously enhancing mass lesion in the right paracolic gutter and right iliac fossa, with calcific focus within.

A 19-year-old woman presented with a 15-day history of severe abdominal pain and a palpable mass, with no history of hematemesis or perforation. Contrast-enhanced computed tomography revealed a well-defined solid lesion with calcification arising from the terminal ileal wall, prompting suspicion of a GIST (Fig. 1). Surgical excision of the terminal ileum, caecum, and appendix was performed for further assessment.

**Figure 2:**
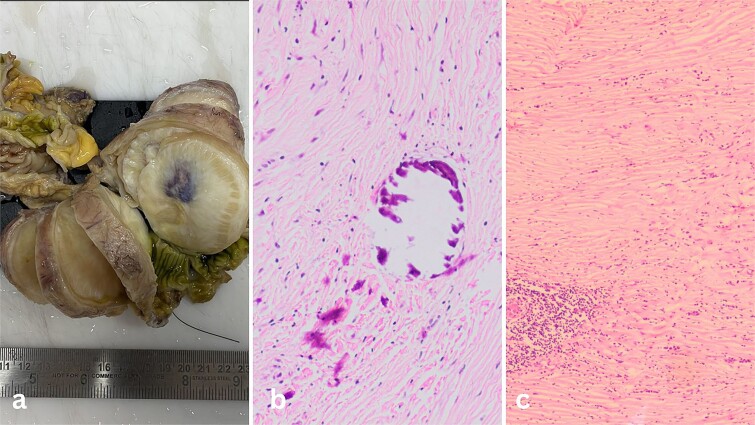
(a) Well-circumscribed mass arising from the wall of the ileum. (b) Calcifications inside the fibrous tumour. (c) Lymphoid aggregates in the bland spindle cell tumor.

The surgical specimen revealed a nodular mass measuring 8.3 × 5.6 × 6.8 cm in the terminal ileum, characterized by its distinct non-encapsulated spherical to lobulated shape with variable calcifications. Sectioning revealed a uniform, partially granular grey-white inner surface ranging in texture from firm to rubbery (Fig. 2a).

Microscopic examination of the tissue sections revealed a predominantly bland spindle cell lesion that was very fibrous and had a large number of dystrophic and psammomatous calcifications (Fig. 1b and c). Inflammatory cells such plasma cells, were present. At first, the features suggested a sclerosing GIST due to the presence of thin-walled prominent blood vessels with perivascular edema and lymphocyte aggregation suggestive of a spindle cell neoplasm. However, the absence of nuclear hypochromasia and minimal cytological atypia called for further investigation.

Immunohistochemical staining was performed to confirm the diagnosis. CD117 (KIT), DOG1, CD34, S100, Beta-Catenin, Desmin, and SMA were negative. This pan-negative IHC profile, combined with the presence of foci of calcification within the lesion, led to the diagnosis of CFT. The Ki67 proliferation rate was low (<1%), confirming the benign nature of the tumor.

## Discussion

CFT is an extremely rare benign tumor originating from fibroblasts, first identified in 1988 by Rosenthal *et al.* as a ‘Childhood fibrous tumor with psammoma bodies’ in peripheral soft tissues [3]. Later, in 1993, Fetsch et al. renamed it calcifying fibrous pseudotumor [4]. This tumor exhibits a wide range of anatomical locations and can mimic various spindle cell tumors. Although its exact etiopathogenesis remains unclear, potential associations include trauma, previous surgeries, and chronic infections [5]. CFT typically affects soft tissues, such as subcutaneous extremities and neck, but can also occur in organs like the pleura, lung, and adrenal gland [6]. It affects individuals of any age, with a median age of 3 years for soft tissue tumors and 43 years for visceral tumors, showing no gender predilection [7]. Gastric CFT is an exceptionally rare mesenchymal benign tumor with unknown pathogenesis, often misdiagnosed as common spindle cell lesions like leiomyoma and gastrointestinal stromal tumors [8]. Macroscopically, CFT appears well-circumscribed, unencapsulated, and solid, with a grey-whitish, gritty texture [9]. Microscopically, it features bland spindle cells, hyalinized collagenous tissue, psammomatous calcifications, and chronic inflammation, including plasma cells and persistent lymphoplasmacytic inflammation [6]. Notably, gastrointestinal tract CFTs differs from soft tissue CFTs by having more perivascular lymphocytes and lymphoid cuffs. Lesional cells express vimentin and Factor XIIIa but not actin, desmin, S100, or cytokeratins, with variable CD34 positivity [10].

Imaging investigations of CFTs generally show a well-circumscribed mass with dispersed calcifications, which is a good representation of their histological nature. When planning surgery, CT and MRI are crucial, as CT is especially helpful in identifying regions of early mineralization that other imaging modalities could miss [1, 11].

The main consideration in the differential diagnosis in our case was GIST and desmoid fibromatosis.

GISTs are the most prevalent mesenchymal tumors in the GI tract, potentially mimicking CFTs in any luminal GI location, particularly in cases where CFTs lack a prominent inflammatory component. GISTs predominantly occur in the stomach, with rare instances reported in the esophagus [11]. Unlike CFTs, GISTs tend to affect younger individuals [9]. Similar to inflammatory myofibroblastic tumor (IMT), GISTs are characterized by the absence of calcifications. Typically, GISTs exhibit c-kit and PDGFRA mutations, accompanied by positive staining for CD117 and DOG1 [12].

-Desmoid fibromatosis is a tumor primarily located in deep soft tissues, notorious for its high recurrence rate. It is distinguished by the proliferation of homogeneous spindle cells in long fascicles within a collagenous stroma. In contrast to CFT, desmoid fibromatosis usually lacks calcifications and displays positive immunohistochemical staining for nuclear beta-catenin, setting it apart from CFT [10].

Several other conditions can present similarly to CFT, making differential diagnosis challenging [10]:

- Calcifying aponeurotic fibroma (CAF): A paediatric condition that mostly affects the hands and feet and is more common in the distal extremities. This is a biphasic fibroblastic tumour that has fibrocartilaginous nodules and spindled fibroblasts. Compared to CFT, CAF has a greater local recurrence rate and is typically less well-circumscribed. The typical location and cartilaginous component aid in differentiating CAF from CFT.

- IMT: Previously believed to indicate a late stage of CFT or occur in conjunction with CFT, this condition is frequently observed in children and young people. As of right now, CFT and IMT are classified differently by the WHO. Histologically, IMT appears as spindle cell proliferation with a noticeable lymphoplasmacytic infiltration; calcifications are rarely observed and the condition is typically more cellular. Unobserved in CFT, an *ALK* rearrangement is seen in up to 50% of IMTs.

- Nodular Fasciitis: A benign and self-limiting cell proliferation of myofibroblasts and fibroblasts. Its rapid growth, high cellularity, and greater mitotic activity can make it resemble sarcomas. It demonstrates extravasated red blood cells, spindle cells with elongated nuclei, inflammatory cells, and mitotic activity along with focal myxoid areas histologically. There is no description of calcifications. In order to differentiate nodular fasciitis from CFT, smooth muscle actin is frequently positive in this condition. A distinctive t(17;22) translocation that results in a *MYH9::UPS6* fusion is commonly seen in nodular fasciitis.

- Solitary fibrous tumor (SFT): SFTs are frequently more cellular with many branching or ‘staghorn’ vessels and lack calcifications. CFT and SFT both express vimentin, whereas CD34 is diffuse positive in SFT but variable in CFT. SFTs express STAT6 and CD99 in contrast to CFTs, due to a rearrangement of the *NAB2::STAT6* gene.

- Leiomyomas share similarities with CFTs in their spindle cell composition and lack of nuclear atypia, and may also exhibit a hyalinized stroma [3]. However, leiomyomas are distinguishable by their characteristic cigar-shaped nuclei and more eosinophilic cytoplasm compared to CFTs. Additionally, leiomyomas typically express markers of smooth muscle differentiation, such as desmin and smooth muscle actin, unlike most CFTs [12].

The primary goal of treating intestinal CFT is to alleviate symptoms like obstruction and intussusception. Segmental resection is the preferred treatment approach, although enucleation has also been performed. CFT is considered a benign entity, and local removal is generally curative. However, rare recurrences have been observed in extra-gastrointestinal lesions [5].

An area of research is the presence of increase in IgG4:IgG ratios in the plasma cell aggregates studied in some CFT cases, pointing to a possible link between CFT and immunoglobulin G4-related illness (IgG4-RD). IgG4-positive plasma cells, a strong lymphoplasmacytic infiltration, and increased serum IgG4 are the hallmarks of IgG4-RD, a fibroinflammatory illness. However, serum IgG levels are not constantly available for assessment, and not all cases of CFT show a raised IgG4:IgG ratio. To better understand the connection between IgG4-RD and CFT, more study is required. [10, 11].
